# Future Trends in Alternative Sustainable Materials for Low-Temperature Thermoelectric Applications

**DOI:** 10.1021/acsaelm.4c00770

**Published:** 2024-07-26

**Authors:** Víctor Toral, Sonia Gómez-Gijón, Francisco J. Romero, Diego P. Morales, Encarnación Castillo, Noel Rodríguez, Sara Rojas, Francisco Molina-Lopez, Almudena Rivadeneyra

**Affiliations:** † Department of Electronics and Computer Science, University of Granada, Granada 18071, Spain; ‡ Department of Inorganic Chemistry, University of Granada, Granada 18071, Spain; ¶ Department of Materials Engineering, KU Leuven, Kasteelpark Arenberg 44, P.O. Box 2450, Leuven B-3001, Belgium

**Keywords:** thermoelectric materials, covalent−organic frameworks (COFs), metal−organic frameworks (MOFs), 2D metal carbides (MXenes), transition-metal chalcogenides (TMDs), black phosporus (BP)

## Abstract

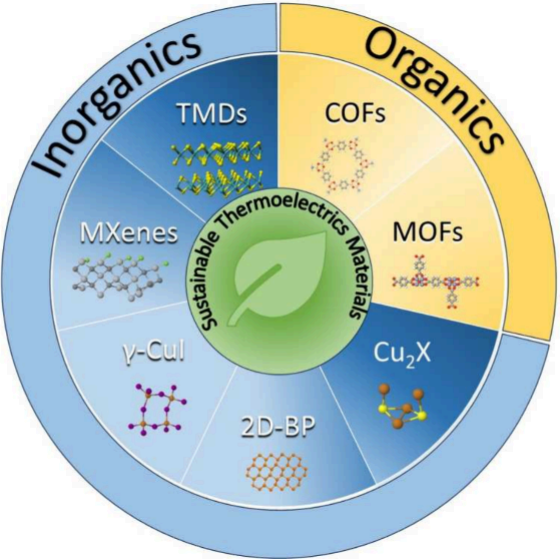

In the evolution of pervasive electronics, it is imperative to significantly reduce the energy consumption of power systems and embrace sustainable materials and fabrication processes with minimal carbon footprint. Within this context, thermoelectric generators (TEGs) have garnered substantial attention in recent years because of the readily available thermal gradients in the environment, making them a promising energy-harvesting technology. Current commercial room-temperature thermoelectrics are based on scarce, expensive, and/or toxic V–VI chalcogenide materials, which limit their widespread use. Thermoelectric polymers partially address this issue, and as such, they have been intensively studied in the field in the past decade. However, less popular materials have recently appeared to respond to the challenges of room-temperature thermoelectrics in terms of sustainability and cost. In this contribution, we comprehensively review the latest advancements in emerging alternative materials with the potential to pave the way for the next generation of sustainable TEGs. This upcoming generation includes flexible and printed TEGs for applications like wearables or the Internet of Things.

## Introduction

In recent years, thermoelectric generators (TEGs) have garnered significant interest as a clean power source owing to their energy-harvesting capability from waste heat. TEGs offer several advantages over other heat harvesters due to their solid-state nature and reliability.^1^

Owing to these characteristics, TEGs have the potential to be used in various applications, including powering nodes for the Internet of Things (IoT) and waste heat recovery in industries and the automotive sector. Among these applications, energy harvesting is particularly suitable for IoT devices because of the low power consumption of IoT nodes and their distributed nature. IoT nodes currently rely on batteries that have limited lifetimes and pose environmental concerns related to manufacturing and disposal.^2^ Moreover, some of the raw materials required for the manufacturing of batteries (Li, Co) are scarce and not readily available in the European Union (EU). In this context, the combination of TEGs with energy storage solutions that can mitigate the blackout moments of TEGs has emerged as an ideal solution for powering IoT devices.^3−6^ In addition to being a power source, TEGs can also be used for fire recognition or temperature sensing.^7−9^ Finally, if TEGs can be made flexible, they will become relevant for wearable devices, either as body heat harvesters or as conformable motion and gesture sensors.^10−14^

**Figure 1:**
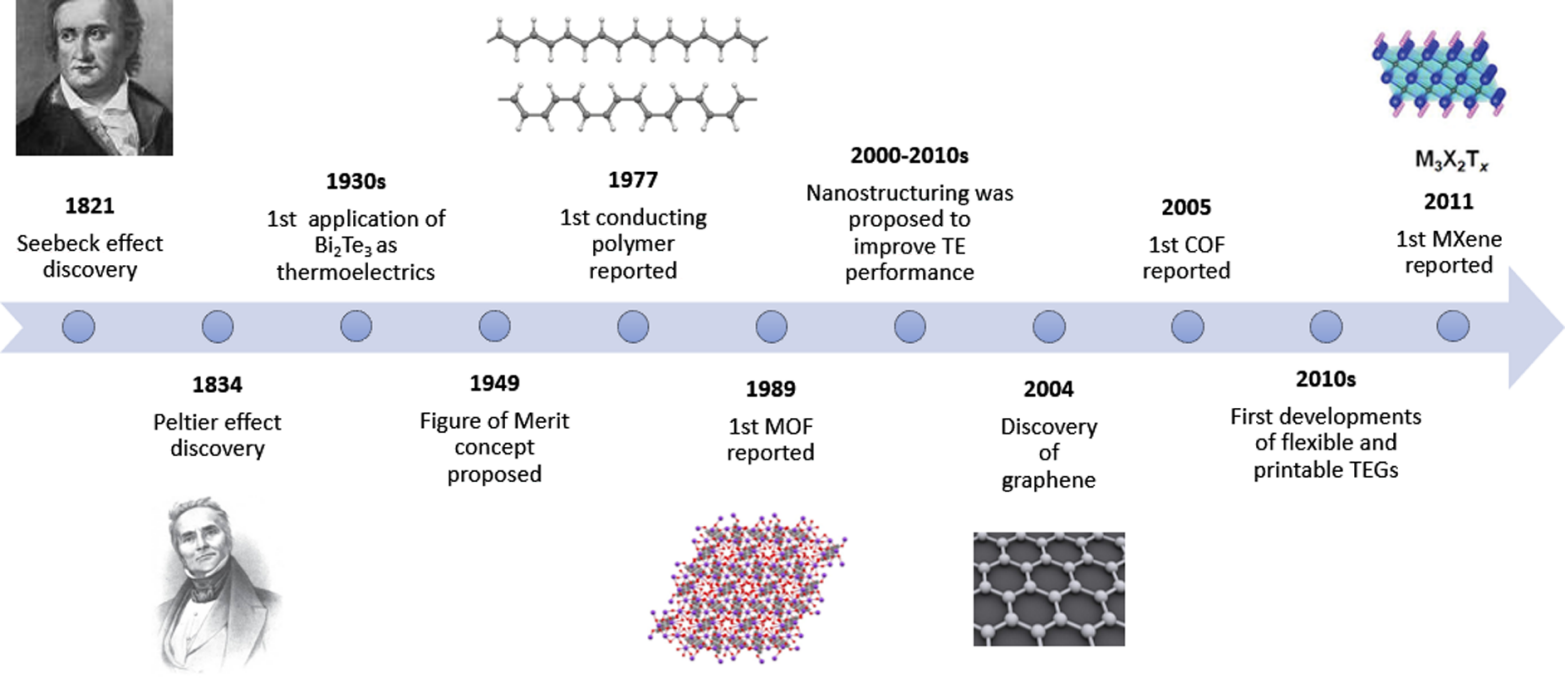
Time line of key developments in thermoelectric technology.

As illustrated in Figure 1, the principles of thermoelectricity have been well-known since the 19^th^ century, and the first TEGs were developed in the early 20^th^ century. Despite this, their use has been less widespread than other available energy harvesting sources, such as solar cells and electromagnetic devices, likely due to their historical low efficiency (typically between 5 and 10%), especially at low temperatures.

Two thermoelectrical (TE) mechanisms are identified in conducting materials: the Peltier–Seebeck effect and the Thomson effect. The Seebeck and Peltier effects are two sides of a reversible process: the Seebeck effect establishes that when a temperature difference is applied to the junction of two different conductors, an electromotive force is generated between their ends. This process is reversible because a temperature difference appears at the ends of the junctions when current is injected through them (Peltier effect). Finally, the Thomson effect describes the heat transfer between a current-carrying conductor subjected to a temperature gradient and its environment. In most practical scenarios, the Thomson effect can be neglected. The performance of TE materials is evaluated through their figure of merit *zT*, defined as shown in eq 1:
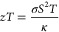
1where σ (S m^–1^) is the electrical conductivity, *S* (V K^–1^) is the Seebeck coefficient, *T* (K) is the absolute temperature, and κ (W m^–1^ K^–1^) is the thermal conductivity. The factor *S*^2^σ is also known as the power factor (PF). In terms of efficiency, a figure of merit *zT* > 1.5 is considered necessary for a competitive TE material.^15^ In this regard, the most competitive thermoelectric materials are inorganic, low-band-gap semiconductors like Bi_2_Te_3_ or PbTe and their alloys, as they can reach *zT* values of up to 2 at around 300 K.^16,17^ Although these materials have traditionally been rigid and difficult to process over large areas, recent efforts have focused on reducing these obstacles.^18^ Organic electronic materials like small molecules and conducting polymers have gained significant attention as viable alternatives to inorganic materials for room-temperature thermoelectric applications. This research is fueled by several advantageous properties, including low material cost, ease of processability via printing techniques, nontoxicity, mechanical flexibility, and low thermal conductivity.^19^ (Semi)conducting polymers, such as poly(3,4-ethylenedioxythiophene) (PEDOT), polyaniline (PANi), polypyrrole (PPY), poly(3-hexylthiophene-2,5-diyl) (P3HT), and poly(2,5-bis(3-dodecylthiophen-2-yl)thieno[3,2-*b*]thiophene) (PBTTT), consist of long conjugated molecular chains packed in films with varying degrees of crystallinity, depending on the specific polymer and its deposition process.^20^ PEDOT, often blended with poly(4-styrenesulfonate) (PSS), is one of the most studied conducting polymers owing to its good thermoelectric properties and superior ambient stability. The results in the literature show PEDOT-based materials with a figure of merit of approaching 0.5 after using different doping strategies to improve the TE performance.^21−23^ Despite being less utilized, PANi, P3HT, and PBTTT have also found applications as performing thermoelectrics.^24^ To generate practical TEGs, both p-type and n-type elements are required. However, all of the mentioned polymers are p-type. Developing performing and chemically stable n-type conducting polymers has proven to be a challenge, although recent chemistries have yielded n-type polymers with values of conductivity (>1000 S cm^–1^) and PF (up to 90 μW m^–1^ K^–2^) approaching those of their p-type counterparts.^25−27^

In addition to conjugated polymers, carbon-based materials represent another relevant technology involving abundant materials that are compatible with flexible and printed devices. An appropriate combination of polymer and carbon nanomaterials leads to composites with much higher electrical conductivity and PFs than neat polymers. Similar to neat polymers, n-type carbon/polymer composites are difficult to fabricate, and the reported performance is significantly lower than that of the p-type. Most n-type composites are a combination of polyethylenimine (PEI) with a carbon-based nanomaterial and can reach PFs of up to 1500 μW m^–1^ K^–2^ while the highest PF for the p-type composite was as high as 3050 μW m^–1^ K^–2^. Recently, densified multiwall carbon nanotube (MWCNT) films have led to ultrahigh PF values of 7250 and 4340 μW m^–1^ K^–2^ for p- and n-type materials, respectively. Unfortunately, compared with neat polymers, the increase in the PF of a carbon/polymer composite comes along a large increase in thermal conductivity. As a result, these composites exhibit a moderate *zT*. Although still better than polymers, carbon/polymer composites are not as performing as traditional inorganic TE materials.^28^

In recent years, several innovative works have demonstrated other interesting sustainable materials based on nontoxic and nonrare elements for their use in thermoelectrics: (i) metal–organic frameworks (MOFs); (ii) covalent–organic frameworks (COFs); (iii) MXenes; (iv) transition-metal dichalcogenides (TMDs); (v) chalcogenides; and (vi) black phosphorus.^29^ Despite not being able to achieve *zT* values as high as those of traditionally used materials, most of these materials are based on abundant/cheap and environmentally friendly elements, some can be printable, and they can operate under mechanical strain, offering a potential future alternative to polymeric and carbon-based thermoelectrics in the development of flexible TEGs. Many reviews have already addressed the TE performance and applications of polymers, carbon-based materials, and their composites.^28,30−32^ This review is different because it focuses on alternative emerging sustainable materials that are suitable for near-room temperature applications (0–100 °C). In this review, we expect to bring attention to new families of green materials that hold potential for room-temperature energy harvesting in low power applications like the IoT and wearables.

## Framework Materials

### Metal–Organic Frameworks (MOFs)

MOFs, also known as porous coordination polymers (PCPs), are a new class of tunable hybrid materials resulting from the self-assembly of inorganic units (e.g., atoms, clusters, chains) and organic polycomplexant linkers (e.g., carboxylates, azolates, phosphonates, among other N- and/or O-donor molecules), which have attracted increasing academic and industrial interest.^33,34^ Compared with other classical porous materials (e.g., activated carbons, zeolites, and silica), MOFs present high structural and chemical versatility together with very high regular porosity featuring different shapes and sizes [pore volume up to 4.4 cm^3^ g^–1^; Brunauer–Emmett–Teller surface area (*S*_BET_) up to 7000 m^2^ g^–1^; pore diameter = 3–98 Å].^35,36^ MOFs possess several characteristics similar to those of organic polymers, including nontoxicity and affordability. In contrast to conjugated polymers, in which the electronic structure (position of the highest occupied and lowest unoccupied molecular orbitals, HOMO and LUMO levels, respectively) is associated with the delocalized π orbitals of the carbon backbone, in MOFs, the presence of transition metal ions introduces new electronic states from the partially filled d or f orbitals of the metal center, which interact with the organic ligands to form the rich electronic structure of the material.

MOFs offer tremendous synthetic and structural versatility through the selection of metal and organic ligands, which allows the modulation of the material electrical and thermal conductivities to optimize *zT*.^37,38^ Furthermore, the high porosity of MOFs presents a unique approach to enhancing thermoelectric performance, as the pores effectively scatter phonons, resulting in reduced thermal conductivity and increased *zT* (eq 1).^39−41^ The long-range crystalline order of MOFs plays a crucial role in promoting high charge mobility, thereby increasing the electrical conductivity without significantly affecting the Seebeck coefficient. Another distinguishing feature of some MOFs is their exceptional ability to adsorb various molecules and nanostructures within their pores. This property enables fine-tuning or even drastic alteration of the materials’ electronic and thermal transport characteristics.^200^ The use of MOFs as TE materials is still in its infancy, and only a few conductive MOFs have been explored in TEGs.

In 2020, Park et al. reported the first 3D MOF (Zn-HAB or [Zn_6_C_24_N_24_], where HAB = hexaaminobenzene; *S*_BET_ = 145 m^2^ g^–1^) with intrinsic thermoelectric properties. By selecting Zn(II) as a tetrahedral metal node, the authors guided the formation of a 3D structure. Unlike d_9_ Cu(II) and d^8^ Ni(II) in 2D conductive MOFs, Zn(II) favors a tetrahedral coordination geometry. Its d^10^ configuration results in a 3D MOF with a p-type semiconductive behavior that provides a Seebeck coefficient of 200 μV K^–1^ and a PF of 3.44 nW m^–1^ K^–2^.^42^

Another interesting MOF is Cu_3_(HHTP)_2_ (HHTP = 2,3,6,7,10,11-hexahydroxytriphenylene). The electrical conductivity of Cu_3_(HHTP)_2_ single crystals was first reported by Hmadeh et al., and it is currently among the best values reported for MOFs (0.2 S cm^–1^).^43^ A current challenge is to process MOFs onto solid supports to facilitate their handling. In this regard, Gonzalez-Juarez et al. studied the electrochemical synthesis of Cu_3_(HHTP)_2_ thin films by anodization and their subsequent transfer to poly(methyl methacrylate) (PMMA), addressing the challenge of the lack of substrate. Thin film deposition improved the thermoelectric behavior, as the Seebeck coefficient increased from −7.24 μW K^–1^ for the bulk materials to −121.4 μW K^–1^ for the thin films, and the PF increased from 2 × 10^–5^ μW m^–1^ K^–2^ to 3.36 × 10^–3^ μW m^–1^ K^–2^.^44^

**Figure 2:**
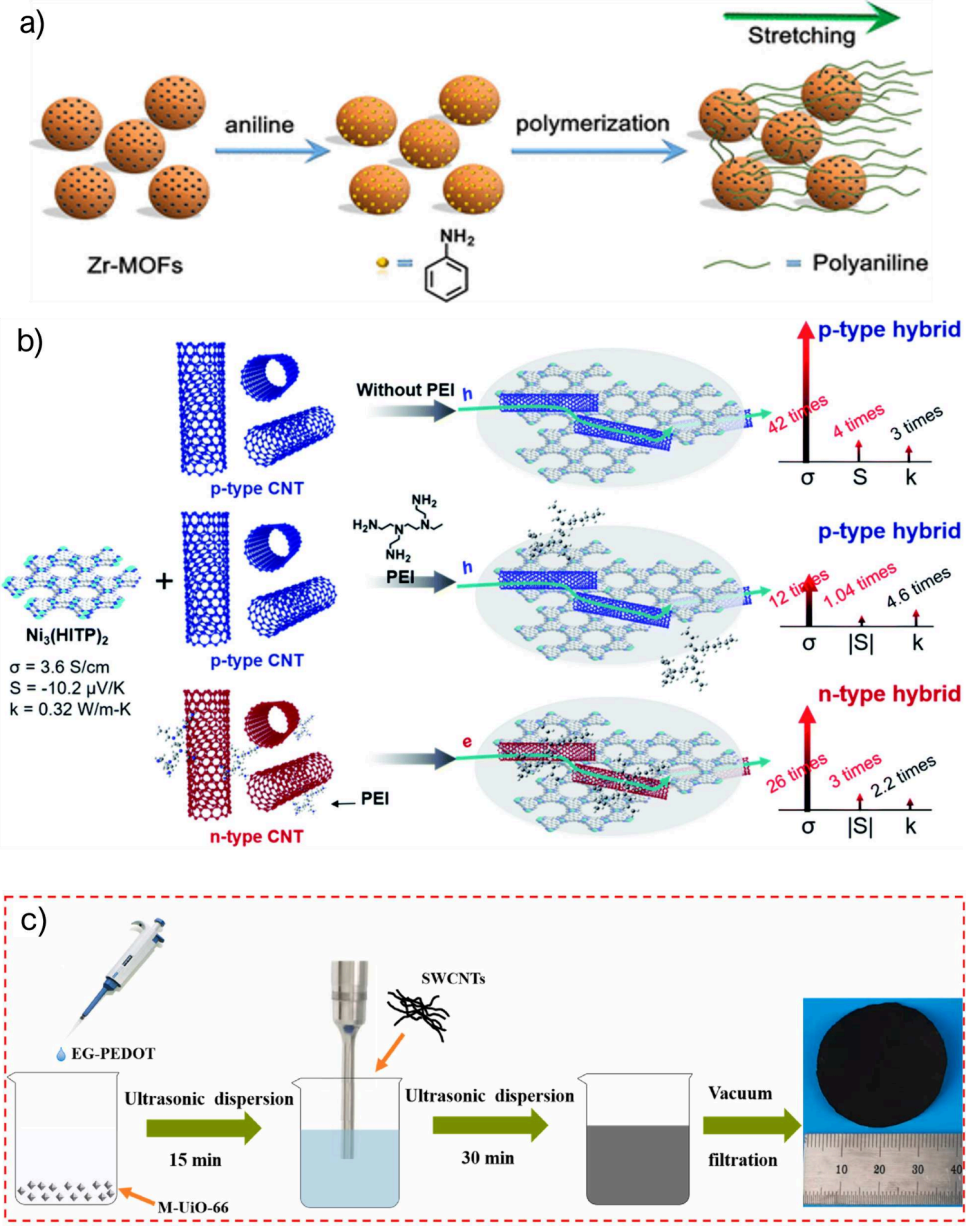
(a) Synthesis of Zr-MOF/polymer composite. Reprinted with permission from ref (45). Copyright 2018 American Chemical Society. (b) Structure of p- and n-type Ni_3_(HITP)_2_-CNTs hybrids. Reproduced with permission from ref (47). Copyright 2021 Royal Society of Chemistry. (c) Fabrication method of a polymer/SWCNT/MOF hybrid. Reproduced with permission from ref (48). Copyright 2022 Elsevier.

MOFs in composites have also been demonstrated to improve the thermoelectric characteristics of pure organic polymers and carbon nanotubes (CNTs). The first example was the polymerization of aniline in Zr-based MOF UiO-66 or [Zr_6_O_4_(OH)_4_(BDC)_6_]nH_2_O (BDC = 1,4-benzodicarboxylate), (*S*_BET_ = 1200 m^2^ g^–1^) using PSS as a dopant.^45^ Following the process shown in Figure 2a, the PANi chains interpenetrated into the UiO-66 structure, resulting in a crystalline PANi with improved electrical conductivity. The composite exhibited an n-type characteristic (Seebeck coefficient of −17.78 mV K^–1^), and both the electrical conductivity and the Seebeck coefficient increased with increasing MOF content. Although the thermal conductivity increased slightly with the MOF content, it did so to a lesser extent than the electrical conductivity, resulting in an enhanced TE performance.

Another elegant MOF-based composite applied in thermoelectrics was reported by Xu et al. In their work, the adsorbed species (free Co^2+^ ions and ligands) found in the pores of a 3D Co-based MOF were exchanged by the conductive ionic liquid 1-ethylpyridinium bromide (EtpyBr) or the photosensitive AgNO_3_, leading to Co-MOF-Br and Co-MOF-Ag, respectively. The p-type conducting polymer PANi was then introduced into the pores of the MOFs, achieving a maximum Seebeck coefficient of 66.5 μV K^–1^ at 400 K and an electrical conductivity of 0.4 S cm^–1^. This resulted in a PF of 17 nW m^–1^ K^–2^. Unfortunately, no value of thermal conductivity was reported.^46^

To further enhance the conductivity of MOFs, they can be combined with CNTs. In particular, Qi et al. hybridized Ni_3_(HITP)_2_ (HITP = 2,3,6,7,10,11-hexaaminotriphenylene) and CNT (30 wt %), leading to a drastic increase in the PF and *zT* values up to 26 μW m^–1^ K^–2^ and 8.77 × 10^–3^, respectively, which is 2 orders of magnitude higher than the *zT* of pristine Ni_3_(HITP)_2_^47^ (Figure 2b). The authors attributed this remarkable improvement to the large increase in both electrical conductivity (from 3.6 to 150 S cm^–1^), and the p-type Seebeck coefficient (from 10 to 40 μV K^–1^), induced by the addition of CNTs. When CNTs (typically a p-type material) were doped with PEI to obtain an n-type composite and mixed with the Ni_3_(HITP)_2_, the PF and *zT* values increased to 9 μW m^–1^ K^–2^ and 3.63 × 10^–3^ (*ca.* 70 times), respectively, in comparison with pristine Ni_3_(HITP)_2_. Both n-type and p-type materials were used to develop a TEG with two TE pairs that could generate up to 67 nW for a temperature difference of 60 K. Despite not having a large output power, this example is a landmark in the development of MOF-based TEGs for actual low-power applications. Although the advantages of blending MOFs and CNTs compared with using CNTs alone remain unclear, this work shows that both chemistries are compatible, which opens the door to future synergies.

Flexible thermoelectric composites can be rationally prepared by mixing MOFs and single-walled carbon nanotubes (SWCNTs). Fan et al. presented a flexible thermoelectric material based on a ternary composite built from acetic acid-modified UiO-66, SWCNTs, and the conducting polymer PEDOT:PSS treated with ethylene glycol (EG-PEDOT:PSS, Figure 2c). The ternary composite films exhibited good flexibility and enhanced thermoelectric performance compared with EG-PEDOT:PSS. EG-PEDOT:PSS rendered the M-UiO-66 moderately conducting, and the SWCNTs bounded all the components as a monolithic flexible film and further boosted the thermoelectric properties, increasing the PF from 0.14 for M-UiO-66/EG-PEDOT:PSS to 27.9 μW m^–1^ K^–2^, when a 40 wt % of SWCNT was added.^48^ Following a similar trend, Chen et al. reported films of SWCNTs@Ni-THT (THT = triphenylenehexathiol). The authors demonstrated how the addition of SWCNTs significantly increased the electrical conductivity of the composite and reduced the Seebeck coefficient. This effect resulted in a noticeable increase in the PF from 0.001 to 98.1 μW m^–1^ K^–2^ with the addition of 4 wt % SWCNTs. A bending study was detailed, showing a low influence of bending on the thermoelectric properties.^49^ Finally, we highlight the work of Xue et al. in the preparation of an SWCNT@MOF flexible composite. Originally, SWCNTs were dispersed in a mixed solution of poly(vinylpyrrolidone) (PVP)/methanol, and Co(NO_3_)_2_ was added to facilitate the Co^2+^ adsorption on SWCNTs surfaces. A mixture of 2-methylimidazole and nano-Co_3_O_4_ in methanol was slowly titrated into the first suspension, leading to an in situ growth of ZIF-67 (Co[mim]_2_ [mim = methylimidazole, *S*_BET_ = 1500 m^2^ g^–1^, pore volume 0.6 cm^3^ g^–1^]) on the SWCNT surfaces. Finally, the precomposite was annealed to obtain a flexible and free-standing film (15 μm thickness) of ZIF-67@CNT composite. With the addition of CNTs and the annealing process, the PF increased from 61.6 to 255.6 μW m^–1^ K^–2^. Furthermore, both the electrical conductivity (825.7 S cm^–1^) and *zT* (0.02) at room temperature were the highest in the experimental data reported so far for MOF-related materials, rivaling those reported for polymeric TEs.^50^

As previously mentioned, MOFs share many features with polymer thermoelectrics. However, their distinctive synthetic and structural versatility offers promising opportunities for optimizing electronic structure via a deliberate choice of metals and ligands to achieve both p-type and n-type materials with high *zT* values. Moreover, such versatility has displayed a tremendous potential for synergy with other materials, such as CNTs and polymers, to deliver composite materials with a high TE performance. The capability to generate n-type MOFs, in contrast to n-type polymers, is noteworthy, especially considering the typical instability of such polymers in the presence of moisture and oxygen.^38,51^ Over 90,000 MOFs have been reported to date, and over 500,000 MOF structures have been predicted, providing an unimaginable number of structures to be studied in TEs.^52^ Considering the early stage of the application of MOF TEs vs conjugated polymers,^53^ this review aims to highlight the potential of MOFs in the field of thermoelectricity.

### Covalent–Organic Frameworks (COFs)

As MOFs, COFs are an exciting new type of crystalline porous polymer constructed exclusively with organic building units (no metal ligand involved) via strong covalent bonds. Compared with conventional organic electronic materials, the covalent bond-supported crystallinity of COFs vastly surpasses the intermolecular force-supported crystallinity of semiconducting molecules/polymers, endowing COFs with superior stability. Furthermore, the porous nature of COFs enables mass transport, which is uncommon for traditional conductive or semiconductive materials that are densely packed.^54^ These characteristics, along with the flexibility of COFs, make them promising candidates for flexible TEGs. The novelty of COFs in the TE field is appreciated in the reduced number of experimental works in the literature, with only ∼10 reports on this topic (most of them theoretical) so far.

**Figure 3:**
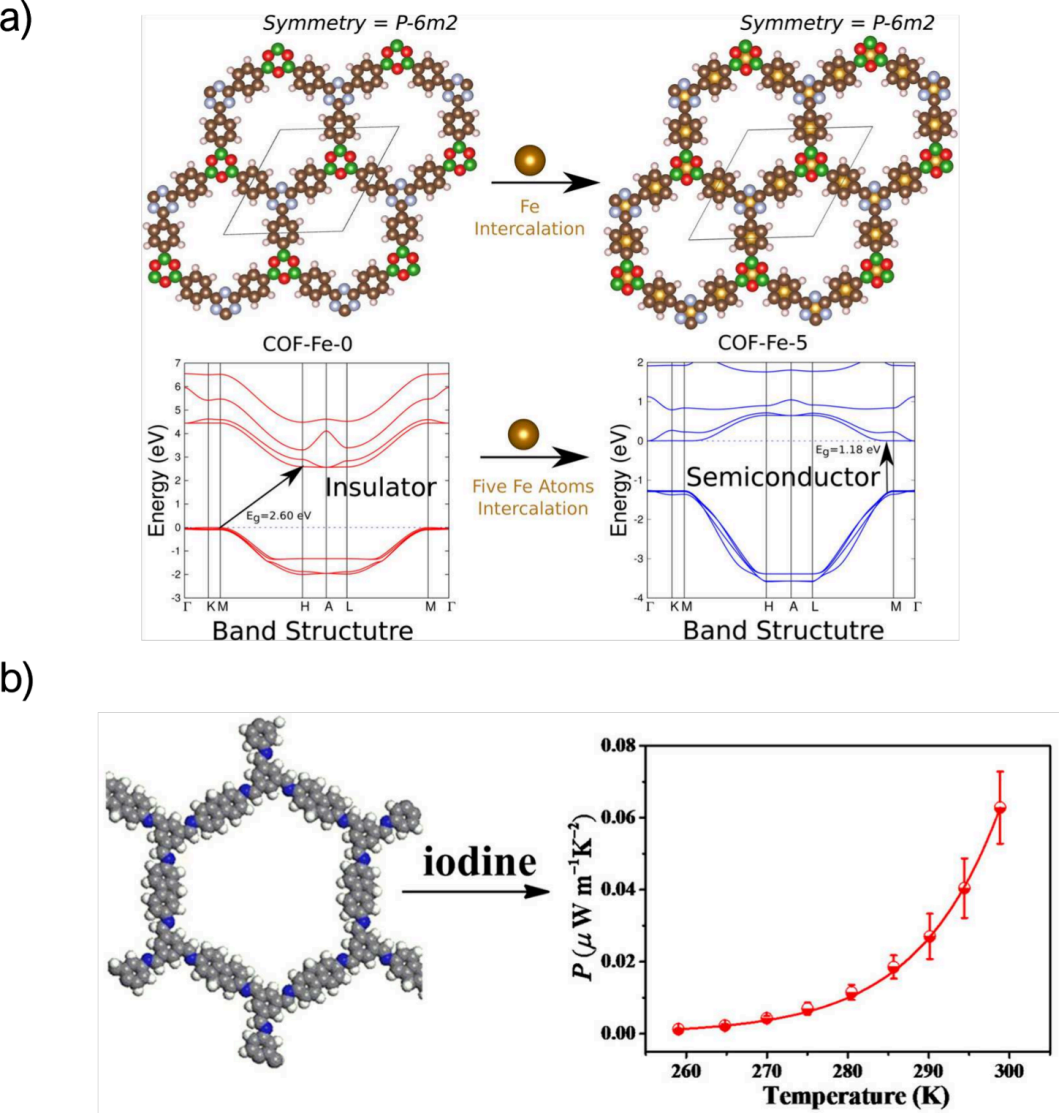
(a) Modification of COF structure with iron atoms. Reprinted with permission from ref (57). Copyright 2017 American Chemical Society. (b) Iodine-doped FL-COF structure and thermoelectric performance. Reprinted with permission from ref (58). Copyright 2017 American Chemical Society.

The first theoretical studies about COF TE properties were focused on their band gap and thermoelectric transport mechanisms, either as raw COFs or through modification of their structure. Chumakov et al. reported for the first time calculations based on density functional theory (DFT) and the Boltzmann transport equation to demonstrate the thermoelectric properties of two phthalocyanine (Pc)-based COFs: NiPc and NiPc-benzothiadiazole (BTDA). As expected, due to the organized arrangement of the Pc units and linkers in these COFs, the transport of charge carriers was facilitated by stacking. In all the compounds, the highly directional character of the p orbitals allowed band-structure engineering and produced a low-dimensional hole transport along the stacking direction of the COF layers. All compounds investigated are indirect semiconductors. Results show promising characteristics for thermoelectric applications, with a maximum theoretical value of *zT* around 0.2.^55^ More recently, three other COFs based on Pc (Cu-Pc, Zn-Pc, and Co-Pc) were studied by Chumakov and Bayram. The calculations showed even better performance than previously reported for Ni-Pc, with *zT* close to 1 for Cu-Pc and Co-Pc and up to 0.65 for ZnPc.^56^ Finally, Pakhira et al. predicted that the electronic properties of COFs can be fine-tuned by adding Fe atoms between two organic layers in the structure. The results presented Fe intercalation as a method to control the band gap of the material and thus the Seebeck coefficient (Figure 3a).^57^

Regarding experimental studies, there are only a couple of experimental works on COF-based thermoelectrics, some of which show promising characteristics. The first study described the condensation of 2,7-diaminofluorene (DAFL) and 1,3,5-triformylbenzene (TFB) to obtain a fluorene-based 2D COF (named FL-COF-1) with high thermal stability and accessible porosity (*S*_BET_ = 1300 m^2^ g^–1^). The open framework was doped with iodine to improve its electrical conductivity. The compressed pellet of I_2_@FL-COF-1 exhibited a Seebeck coefficient of 2450 μV K^–1^ and an electrical conductivity of 1 × 10^–4^ S cm^–1^, which resulted in a PF of 0.063 μW m^–1^K^–2^ (Figure 3b).^58^ This strategy was also used by Wang et al., in whose work, the I_2_ doping of a metal-Pc-based pyrazine-linked 2D COF (named as ZnPc-pz-I_2_), led to a notable improvement in the Hall mobility from 5 to 22 cm^2^ V^–1^ s^–1^, making these materials good candidates for TE applications.^59^

In the third study, the approach followed to prepare n-type COF semiconductors was direct polycondensation of conventional p-type knots with an n-type indigo linker [6,6′-*n*,*n*′-(2-methyl)isoindigovibronic acid or MIDA] to form nonconjugated tetragonal and hexagonal two-dimensional polymeric frameworks. The authors selected knots from a well-known HHTP with a well-established pi-stacking structure, p-type semiconducting behavior, and phthalocyanine (Cu-Pc) as a typical 18-electrons macrocycle with a well-defined planar structure. The resulting HHTP-MIDA-COF and CuPc-MIDA-COF were planar in conformation and showed flattened frontier molecular orbital levels, which enabled electrons to move along the nonconjugated polymeric backbones. Furthermore, the hall resistance was measured to determine the mobility and carrier type. The authors obtained a high electron mobility of 8.2 cm^2^ V^–1^ s^–1^, which makes these materials promising candidates for n-type thermoelectrics.^60^

Similar to MOFs, COFs have very versatile structures that allow to tune their thermoelectric characteristics. However, their application in the TE field has been barely studied in the literature yet; thus, there is still a long way to make them competitive in terms of TE performance.

### Metallic Chalcogenides

In addition to well-known group V–VI chalcogenides, metal chalcogenides like Ag chalcogenides and Cu chalcogenides have emerged as promising TE materials. In particular, the latter compounds have attracted intensive interest recently. They have the formulation Cu_2_X, where X denotes Se or S. These compounds are p-type semiconductors that exhibit exceptional electrical and thermal transport characteristics and thus have a high figure of merit at medium to high temperatures. The compounds with Se showed better performance and were more studied in the literature than those with S. However, S compounds are more suitable for this review because S is less toxic and more abundant than Se. This lack of toxicity contrasts with the IV–VI and V–VI chalcogenides, which contain Pb and Sb.^61−64^ Furthermore, Cu is less scarce and cheaper than Pb, Sb, or (especially) Bi, which are typically used in IV–VI and V–VI TEs.^65^

Recent works on Cu_2_S show that it can reach high *zT* and PFs at high temperatures. The first attempt to print Cu_2_S was in 2019 by Burton et al., who fabricated a 3D-printed TE with a *zT* of 0.63 at 966 K. This is a low value compared with other works based on bulk Cu_2_S,^67^ but the study presents the advantage of a printable and scalable method for TE materials.^66^ More recently, Yue et al. achieved a *zT* close to the highest value reported for Cu_2_S^67^ using a simple fabrication method. They described a hydrothermal process to develop a micro/nano Cu_2–*x*_S composite, which reached a *zT* value of 1.1 at 773 K thanks to its low thermal conductivity (0.69 W m^–1^ K^–1^). These results demonstrate that Cu_2_S compounds are perfect candidates to fabricate TE devices with a good performance.

Further improvement of the performance of Cu_2_S can be possible using dopants, as studied by Zhang et al. In this work, the authors tested several dopants, including In, Cd, Zn, Sn, and Pb. The doped composites were fabricated using a colloidal solution of nanoparticles that, once doped, were dried and annealed at 400 °C; finally, the composites were hot-pressed to form pellets. From the results obtained, we concluded that Pb is the most interesting dopant from a performance perspective because the Pb-doped Cu_2_S pushes the *zT* to 2.03 at 900 K. This is the highest *zT* reported for Cu_2_S. However, the use of toxic Pb is a limiting point from a sustainabilty standpoint.^69^ Although the temperature at which these impressive results are achieved limits the application of these sustainable and abundant materials to specific scenarios, such as the automotive industry^68^, future developments might increase their performance at lower temperatures, which is a relevant aspect for pervasive electronics. This prospect makes it worthwhile to closely monitor the progress in the field over the next years. Other authors have tried to exploit the use of Cu_2_S at low temperatures by blending it with polymers. This is the case of Zhao et al., who studied the influence of Cu_2_S in PEDOT:PSS screen-printed TE films. The composite was characterized at content ratios of 1:1.1 to 1:1.4 of Cu_2_S and PEDOT:PSS, respectively. The results show that the conductivity increased with PEDOT:PSS content, whereas the Seebeck effect was reduced. Consequently, the change in PF was not significant among the different concentrations; the highest PF was 20 μW m^–1^ K^–2^ for a 1.2 ratio, while the lowest was 18 μW m^–1^ K^–2^ for a 1.1 ratio. The authors demonstrated the utility of this material by fabricating a TEG using Ag_2_Se for the n-type legs. This device was able to generate up to 160 nW for a temperature difference of 35 K, which is modest in comparison to other Cu_2_S-based devices, but probably because the top performance of Cu_2_S was obtained at nonpractical high temperatures (around 900 K).^70^

### γ-CuI

γ-CuI is a transparent p-type semiconductor that has been extensively used as a transparent electrode in solar cells, displays, and light-emitting devices. The applications of γ-CuI in the thermoelectric field have been also studied. This material is interesting because it is nontoxic. γ-CuI has a wide band gap (3.1 eV) and reduced thermal conductivity as iodine is a heavy element.

**Figure 4:**
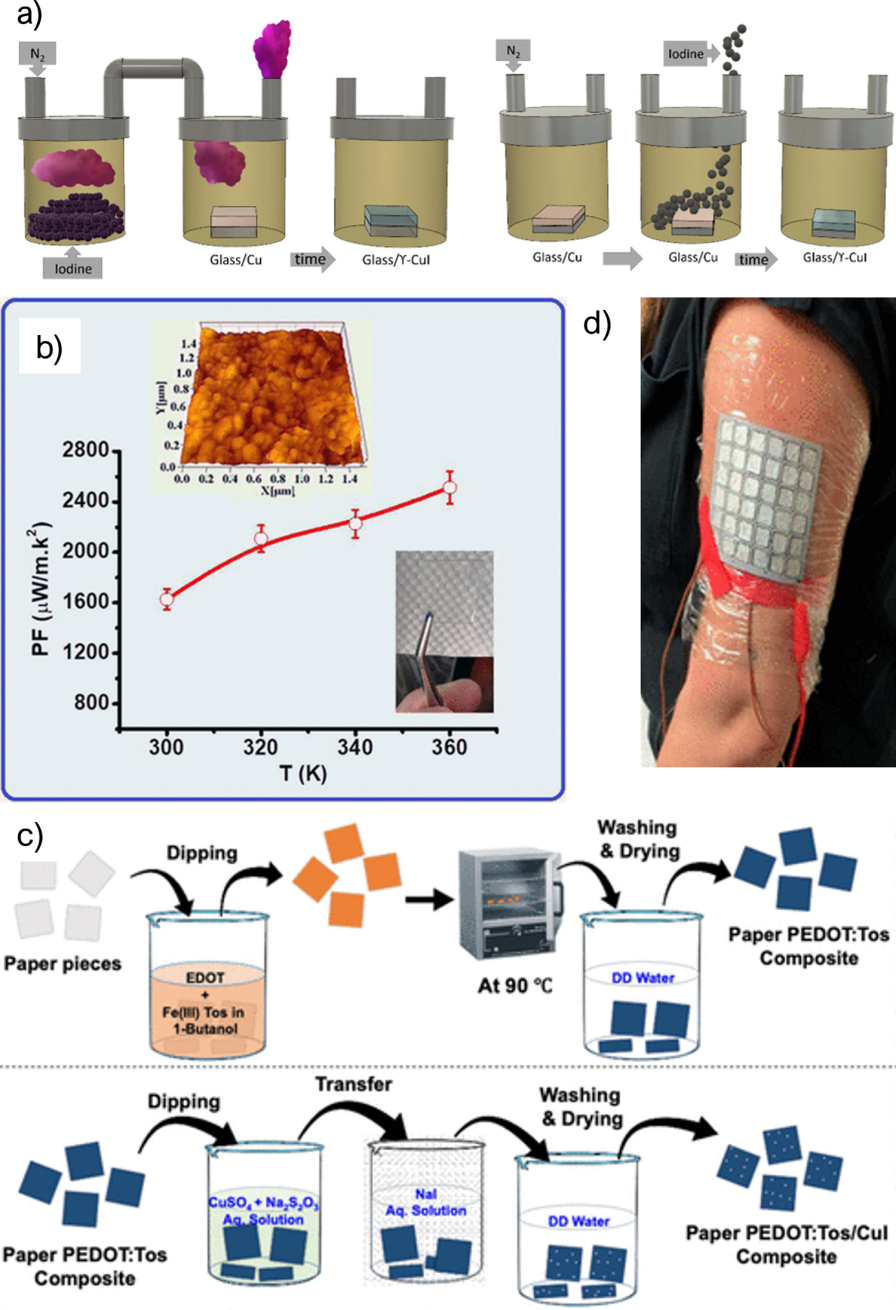
(a) CuI fabrication processes using vapor or solid iodination. Reproduced from ref (72). Available under a CC-BY license. Copyright 2018 Springer Nature. (b) PF and details of the CuI TE fabricated by Almasoudi et al. Reprinted with permission from ref (73). Copyright 2022 American Chemical Society. (c) Process used to prepare the PEDOT:Tos/CuI composite. Reprinted with permission from ref (75). Copyright 2021 American Chemical Society. (d) Example of the use of a wearable TEG based on a composite of PEDOT:Tos and CuI. Reprinted with permission from ref (75). Copyright 2021 American Chemical Society.

Yang et al. studied the influence of carrier concentration on thin films of γ-CuI fabricated via reactive sputtering. Their results showed a maximum *zT* of 0.21, a carrier concentration of 10^20^ cm^–3^, and a PF of 375 μW m^–1^ K^–2^ at 320 K. Furthermore, the authors studied their behavior as a one-leg TEG, achieving an output power of 8 nW at a difference of temperature of 10 K.^71^ More recently, Morais Faustino et al. presented three fabrication methods for CuI (Figure 4a): thermal evaporation of CuI powder, vapor iodination of Cu films. The best result was achieved for solid iodination and corresponded to a PF of 470 μW m^–1^ K^–2^. Finally, they developed a TEG using gallium-doped zinc oxide (GZO) as the n-type leg. With this structure, the authors achieved an output power of 0.45 nW at a temperature difference of 13 K. This value of output power is lower than expected for the high PF measured.^72^ In 2022, Almasoudi et al. used the pulsed laser deposition to CuI. With this method, the authors achieved an outstanding PF of 2400 μW m^–1^ K^–2^ and a *zT* of 1.12 at 360 K, as shown in Figure 4c. Furthermore, the resulting film is flexible and transparent, making it a perfect candidate for wearable applications^73^

Other authors like Salah et al. and Maji et al. studied the possibility of using other elements to improve the performance of CuI. First, Salah et al. studied several possible dopants, including metals, semimetals, and rare earths. The best result was obtained by doping CuI nanoparticles with 0.05 mol % Tb, which increased the *zT* from 0.05 for pristine CuI to 0.28 at 420 K.^74^ Another innovative strategy followed by Maji et al. was to fabricate a composite of PEDOT:Tos and CuI on a paper substrate following the process illustrated in Figure 4d. Compared with neat PEDOT:Tos, the addition of CuI increased the Seebeck coefficient from 63 to 225 μV K^–1^. Finally, the authors developed a device composed of 36 legs of this TE material connected in series (Figure 4b) that could produce up to 57.9 nW from human body heat (at a temperature difference of around 4 K).^75^

## 2D Inorganic Materials

### MXenes

MXenes are layered transition-metal carbides, carbonitrides, or nitrides discovered in 2011.^76^ MXenes are obtained from layered ternary materials known as M_*n*+1_AX_*n*_ or MAX phases, which are a large group of layered hexagonal compounds, where M is a transition metal, A is an A-group element (mostly groups 13 and 14), X is C or N, and *n* is 1–3. When the A-layers are chemically etched, the result is weakly bound stacks of 2D sheets with a M_*n*+1_X_*n*_T_*x*_ composition, where T_*x*_ represents the surface termination.^77^ These materials are 2D materials with promising applications, most of them in the energy field as storage elements, electromagnetic shielding, and, more recently, also as TE.^78^ MXenes have the advantage of being nontoxic and abundant materials, in contrast to traditional inorganic materials like group V–VI chalcogenides. Moreover, recent progress in process scalability and shelf life has suggested their viability for industrial applications.^79^

**Figure 5:**
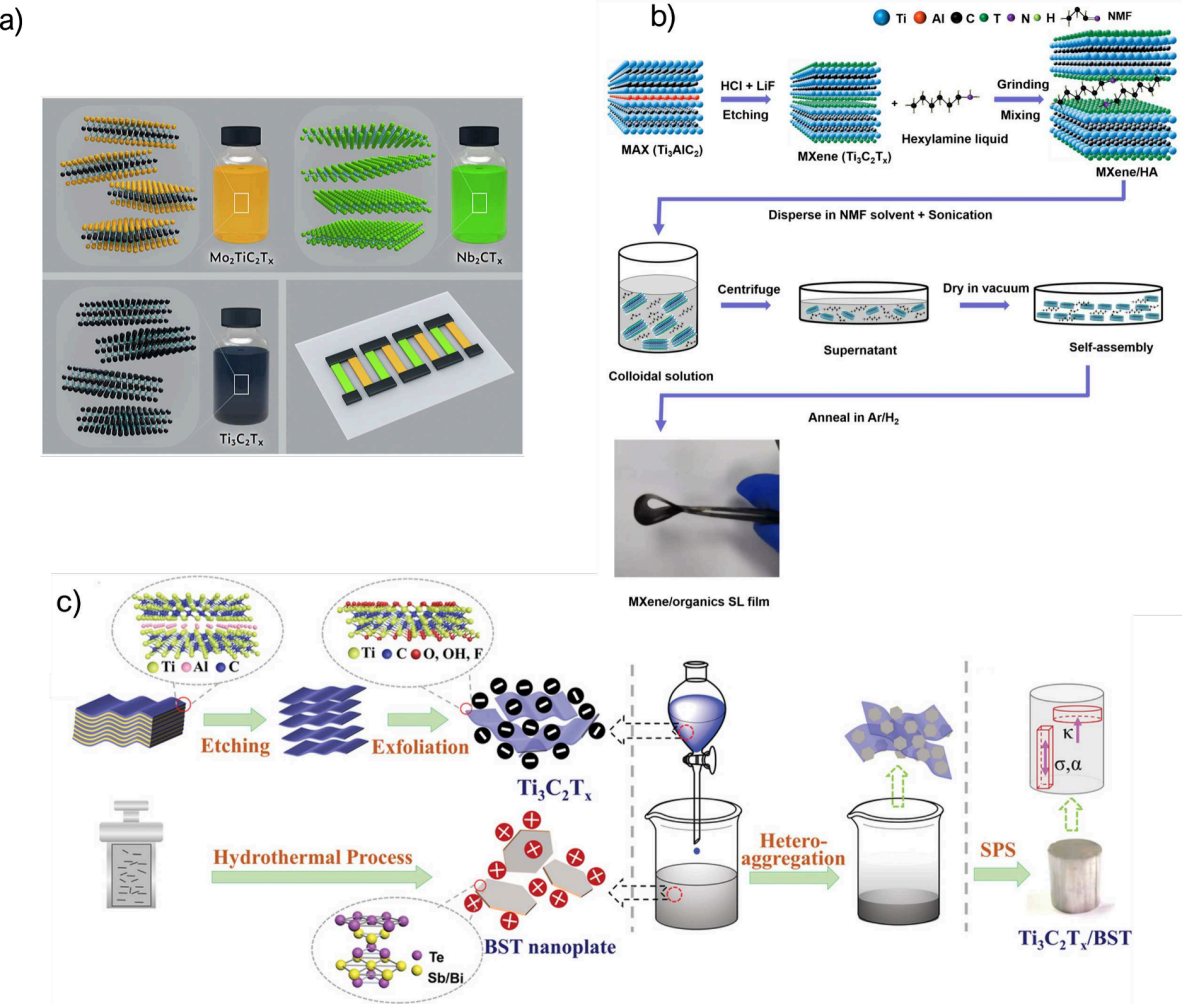
(a) All MXenes used in the TEG fabrication. Reprinted with permission from ref (80). Copyright 2022 Elsevier. (b) Fabrication method of MXene and an organic superlattice flexible thermoelectric compound. Reprinted with permission from ref (82). Copyright 2022 American Chemical Society. (c) Fabrication methods of BST and MXene thermoelectric materials. Reprinted with permission from ref (87). Copyright 2019 Wiley.

Very recently, MXenes based on Mo_2_TiC_2_T_*x*_ and Nb_2_CT_*x*_ have been used for thermoelectricity featuring high PF, as demonstrated by Huang et al.^80^ The authors developed a full MXene TEG (Figure 5a), where the n-type leg was made of Mo_2_TiC_2_T_*x*_, the p-type leg of Nb_2_CT_*x*_, and Ti_3_C_2_T_*x*_ was used for contacts. The TEG was fabricated using a combination of screen printing, poly(dimethylsiloxane) (PDMS) masking, and dropcasting. With these materials, the authors reached a PF of 13.26 μW m^–1^ K^–2^ for the n-type MXene and 11.06 μW m^–1^ K^–2^ for the p-type. The final device provided up to 35 nW for a temperature difference of 30 K using 20 TE pairs, which was a low value compared with other related works; for example, Qi et al. achieved 65 nW using only two pairs. However, the work of Huang et al. is remarkable due to the achievement of an n-type TE material, which is more challenging to obtain than p-type materials.

The TE performance of pure MXenes can be enhanced in several ways. Liu et al. demonstrated that through strong basic treatment (KOH under hydrothermal conditions), the TE behavior of Ti_3_C_2_T_*x*_ can be improved. Through this process, some F-terminal groups of the MXenes were replaced by K, increasing the electronic band gap of the material. This resulted in a significant improvement in the Seebeck coefficient from 6.6 to 20 μV K^–1^. On the other hand, the electrical conductivity was reduced as the KOH content in the reaction increased. An optimal point at which the PF was maximized to 45 μW m^–1^ K^–2^ was found when the KOH concentration was 12 mM. Unfortunately, the authors did not provide any information on thermal conductivity, making it impossible to determine the figure of merit. Nonetheless, a flexibility study was presented, reporting variations in the PF of less than 10% after 1000 bending cycles.^81^ Following a different strategy, Wang et al. enhanced the carrier mobility and density of a Ti_3_C_2_T_*x*_-organic superlattice using the process shown in Figure 5b.^83^ In this work, MXene was combined with hexylamine (HA), resulting in a flexible film with n-type thermoelectric behavior. When annealed at 150 °C, the composite exhibited a PF of 33 μW m^–1^ K^–2^.

Sarikurt et al. investigated the TE properties of oxygen-functionalized MXenes. A theoretical analysis was employed to examine the thermal transport and thermoelectric characteristics of various MXenes, specifically those with the composition M_2_CO_2_ (where M = Ti, Zr, Hf, Sc), considering two distinct crystalline structures. The relaxation time approximation was used to predict the thermoelectric characteristics of MXenes under both n-type and p-type doping conditions. The results revealed a notable theoretical *zT* value of 1 at moderate carrier densities across all examined crystalline structures, with particularly high Seebeck coefficients observed for Zr_2_CO_2_ and Hf_2_CO_2_. This suggests that oxygen-functionalized MXenes exhibit promising potential as thermoelectric materials.^84^

Following a similar trend to that of MOFs, the use of MXenes in the thermoelectric field has led to the preparation of composite materials to improve their thermoelectric performance. Chalcogenides and other inorganic compounds (i.e., ZnO) are common materials used in the preparation of MXene composites for TEG because their performance can be improved using MXenes. One example is the study of Guo et al., in which an improvement of 78% in *zT* is achieved when adding Mo_2_CT_*x*_ to Bi_2_Te_3_.^85^ Other works report the addition of Ti_3_C_2_T_*x*_ to bismuth antimony telluride (BST) compounds, leading to an improvement of up to 48% in *zT* (Figure 5c).^86,87^ More examples of enhanced thermoelectric properties are the composites based on the chalcogenides GeTe, SnSe, and SnTe with Ti_3_C_2_T_*x*_ achieving exceptional PF values up to 2000 μW m^–1^ K^–2^.^88−90^ Although these chalcogenides include rare and/or toxic elements, which are not the main focus of this review, these examples are still interesting because they illustrate the potential of mixing MXenes with benchmark materials.

Indeed, the thermoelectric performance of more sustainable inorganic compounds other than chalcogenides can be further improved using MXenes. The work by Yan et al. demonstrated the strategy of depositing ZnO layers on Ti_3_C_2_T_*x*_ films by atomic layer deposition (ALD). With this method two effects were observed: the Seebeck effect was magnified by the increased mobility of high-energy carriers, and the thermal conductivity was reduced. Thus, the overall *zT* was highly enhanced, reaching a value of 1.8 × 10^–3^ at 625 K, which, despite being a low value, was four times higher than that of pristine MXene films.^91^ In a similar study, the thermoelectric characteristics of Cu iodide were enhanced by blending it with Ti_3_C_2_T_*x*_ in a composite. The results show that a boost in carrier density coming from Ti_3_C_2_T_*x*_ produced an electrical conductivity improvement. Adding only 5 vol % of MXenes improved the figure of merit b five-fold compared to pristine CuI and led to a PF value as high as 100 μW m^–1^ K^–2^ at 400 K.^92^

Other less studied materials used in the preparation of MXene-based TE composites include SWCNTs, organic polymers, and perovskites. One interesting work was reported by Wei et al. on the preparation of a p-type structure composed of SWCNTs and Ti_3_C_2_T_*x*_. The best performance was achieved with 10 wt % of MXene. SWCNT@Ti_3_C_2_T_*x*_ reached a PF value of 203.23 μW m^–1^ K^–2^ at room temperature, and a *zT* 20-fold higher than pristine SWCNT.^93^ Another example of SWCNT@MXene composite was presented by Ding et al. This time, the prepared composite was a sandwich structure of Ti_3_C_2_T_*x*_/SWCNT/Ti_3_C_2_T_*x*_, which enhanced the electrical conductivity of the material, and thus, the PF, which was increased by 25-fold (from 3.12 to 77.9 μW m^–1^ K^–2^) compared with that obtained with neat Ti_3_C_2_T_*x*_.^94^ In the use of polymers in the preparation of MXene-based composites, it should be noted the work of Guan et al. In this work, Ti_3_C_2_T_*x*_ was included in PEDOT:PSS films, generating an energy-filtering effect that increased the Seebeck coefficient of the compound. This filtering effect was observed only at MXene concentrations under 33 wt %, as this ensured that the MXene sheets were not connected between them. Through this mechanism, the authors reported an increase in the Seebeck coefficient from 23 to 57.3 μV K^–1^ while the electrical conductivity was reduced from 800 to 150 S cm^–1^, thus increasing the PF from 40 to 155 μW m^–1^ K^–2^.^95^ Finally, Ti_3_C_2_T_*x*_ MXenes also improved the n-type oxide perovskite SrTi_0.85_Nb_0.15_O_3_ (STN) thermoelectric properties. Thanks to the inclusion of MXenes in the STN, the electron mobility was enhanced, and the conductivity of the compound was significantly increased. As a result, this work achieved an outstanding increase of *zT* by 7-fold, which reached a value of 0.9 at 900 K. The PF reached 3000 μW m^–1^ K^–2^ at 500 K. Furthermore, the authors presented a device prototype with four legs of STN + 1 wt % MXene that generated up to 38 mW at a temperature difference of 713 K. This output could be sufficient to power a sensor node without a battery or with the backup of a supercapacitor.^96^ However, these impressive values were achieved at very high temperatures and temperature difference, which limits the applicability of this material in the field of pervasive electronics.

### Transition-Metal Dichalcogenides (TMDs)

TMDs are 2D materials with a formulation of MX_2_ based on a chalcogenide (X) and at least one electropositive element (M). These materials have garnered a lot of interest in recent years due to their interesting electrical properties, including thermoelectricity.^97^ TMDs show a high PF due to their high Seebeck coefficient and high electrical conductivity; however, their figure of merit is limited by their high thermal conductivity.^98^

Among the most promising TMDs for thermoelectricity are materials based on Mo and W.^99^ Several theoretical works have reported on their TE properties,^100−103^ such as the work developed by Ouyang et al., providing the calculated highest performance of MoS_2_/MoSe_2_ hybrids nanoribbons with a figure of merit of 7.4 at 800 K; or the first-principles calculations carried out by Purwitasari et al., where Tc-based TMDs can reach a figure of merit of 1.8 at 1200 K.^104^

**Figure 6:**
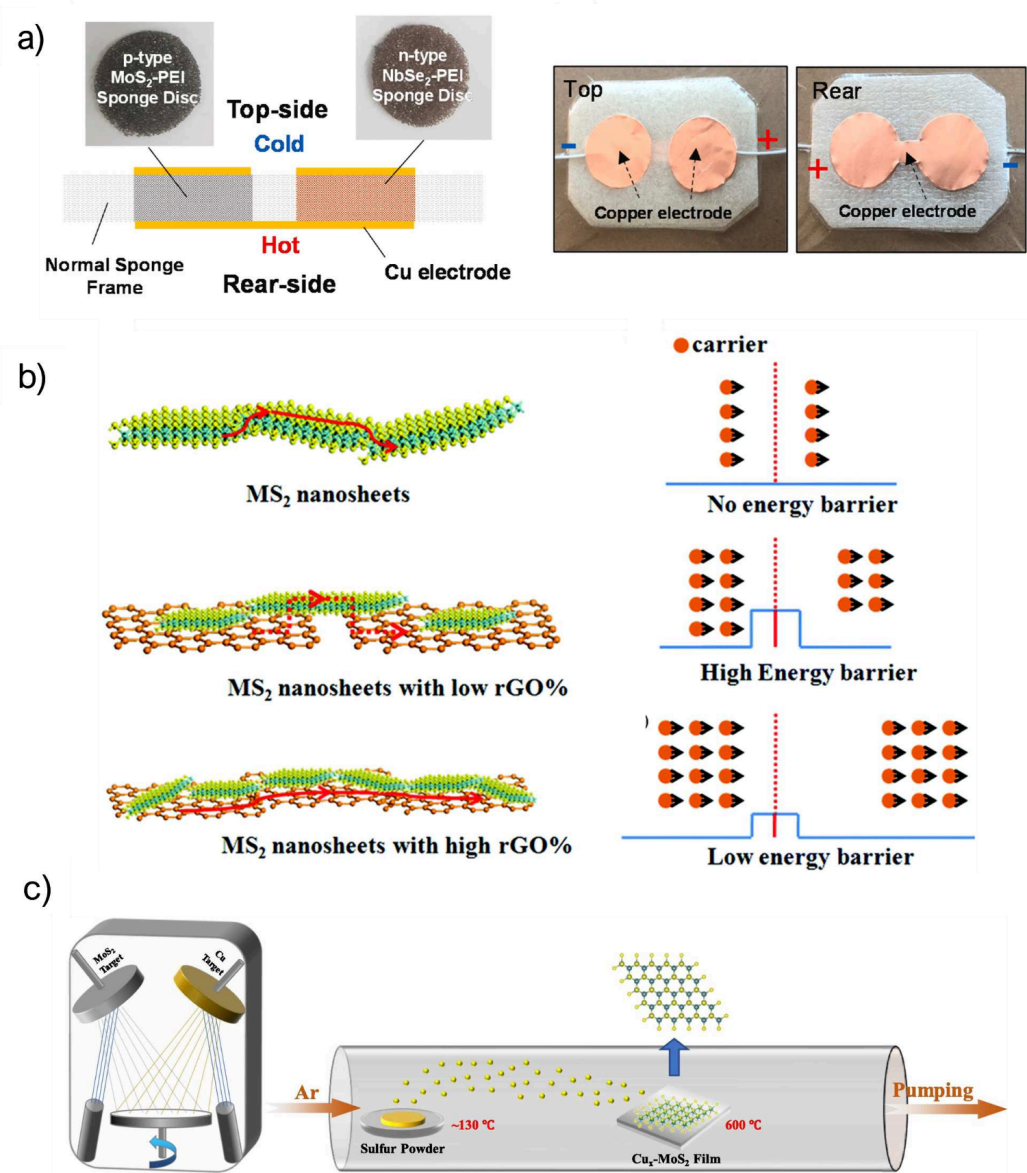
(a) Deformable TEG based on n- and p-type TMDs. Reprinted with permission from ref (107). Copyright 2023 Elsevier. (b) Filtering effect from adding rGO to MS_2_. Reprinted with permission from ref (113). Copyright 2017 Royal Society of Chemistry. (c) Fabrication process of Cu-MoS_2_ hybrid films. Reprinted with permission from ref (115). Copyright 2023 Elsevier.

On the other hand, there are a few experimental works on thermoelectrics based exclusively on TMDs, since most include also toxic materials like Se or Te.^105,106^ Nghia et al. showed a TEG based on p-type MoS_2_ and n-type NbSe_2_. In this work, the authors used PEI and a melamine sponge as substrates to fabricate a flexible device to be adhered to the skin, obtaining energy from body heat. The device is presented in Figure 6a. The results show a figure of merit of 5.4 × 10^–3^ and a PF of 0.537 μW m^–1^ K^–2^ for the p-type material and a figure of merit of 1.36 × 10^–3^ and a PF of 0.035 μW m^–1^ K^–2^ for the n-type material. Although the results obtained were not exceptionally high, their application closely aligns with a wearable TEG.^107^ All of these studies exhibit results that are clearly worse than theoretical studies, which means that there is still plenty of room for improvement.

A recently studied n-type material is TiS_2_ monolayers. While layered TiS_2_ bulk was already known, and the intercalation of transition metals on TiS_2_ with very high performance (PF = 37.1 μW m^–1^ K^–2^ and *zT* = 0.16 at 300 K) was reported in 2011, it was not until then that monolayers were suggested as a promising material for thermoelectricity at low temperature.^108,109^ More recently, Li et al. demonstrated by first-principles calculations that the performance of TiS_2_ can be enhanced by applying strain to the material. This point was experimentally confirmed by Salah et al., who were able to fabricate TiS_2_ pellets with a high TE performance near room temperature. The authors demonstrated that the strain generated by contraction at low temperatures increased the output power of a single leg by six times and achieved a PF of 540 μW m^–1^ K^–2^. Despite these exciting results, the authors reported only a modest value of *zT* = 0.04 at temperatures above room temperature (up to 100 °C).^110,111^ Another strategy that yielded good results in enhancing the performance of neat TiS_2_ is microstructural texture engineering. Gu et al. realized an ethanol-based pulverization process followed by Spark Plasma Sintering to produce highly textured and small-grain ceramics. Compared with the pristine synthesized powder, an enhanced PF was driven by the high texture and a reduced thermal conductivity by the small grain size. These improvements resulted in an increase of 75% in *zT* (from 0.4 to 0.7) and 65% in PF (from 1 to 1.7 mW m^–1^ K^–2^).^112^

Similar to the other materials reviewed in this paper, TMDs can also be used to fabricate TEG composites. The composites discussed in the literature, as anticipated from theoretical studies, predominantly involve MoX_2_ TMDs. For instance, Wang et al. investigated the impact of reduced graphene oxide (rGO) on the thermoelectric properties of MoS_2_ and WS_2_. These hybrid materials exploit the junction effect between rGO and TMDs, creating an energy barrier that filters low-energy carriers, as represented in Figure 6b. This resulted in an enhancement of the Seebeck coefficient. Remarkably, the electrical conductivity also increased, which consequently enhanced the PF. The authors achieved a PF of 15.1 μW m^–1^ K^–2^ for the MoS_2_ composite and 17.4 μW m^–1^ K^–2^ for the WS_2_ composite, marking a 1.5 times improvement compared to pristine TMDs. These compoite materials exhibited *zT* values of 0.022 for MoS_2_ and 0.025 for WS_2_.^113^

Furthermore, several examples exist in which metallic particles have been employed to enhance the TE performance of pristine TMDs. For example, the TE performance of MoS_2_ can be further improved through decoration with Ag nanoparticles, as demonstrated by Li et al.^114^ The Ag@MoS_2_ composite presented in this work attained a PF of 30.3 μW m^–1^ K^–2^. Cu is another candidate for doping MoS_2_, as demonstrated by Xin et al. In this study, MoS_2_ was doped with Cu by magnetron sputtering followed by chemical vapor deposition (CVD) (Figure 6c). Finally, the compound was annealed at 873 K. The PF of this composite was 1.25 μW cm^–1^ K^–2^, and the figure of merit was 0.137 at 450 K, which improved the *zT* by an order of magnitude compared to pristine MoS_2_.^115^ More recently, Yang et al. achieved an outstanding TE performance in MoS_2_ by adding aluminum. The Al@MoS_2_ compound exhibited a PF of 122 μW m^–1^ K^–2^, nearly double that the one of neat MoS_2_.^116^

TiS_2_ have been also extensively used along with polymers. For example, Wan et al. manufactured an n-type thermoelectric by intercalating phenylammonium between layers of TiS_2_. With this structure, the new materials maintained the PF while reducing 7 times the thermal conductivity, which resulted in a *zT* of 0.28 at 370 K (3 times higher than a single TiS_2_ crystal).^117−119^ Another strategy presented by Wang et al. was the combination of TiS_2_ with fullerene. In this study, the authors developed a method to intercalate fullerene between TiS_2_ layers. The composition of the hybrid films was optimized to maximize the thermoelectric performance at a 1 wt % of C_60_. At this composition, the hybrid films achieved an outstanding *zT* of 0.3 and a PF of 375 μW m^–1^ K^–2^ at 400 K. Furthermore, they fabricated a TEG with PEDOT:PSS as the p-type legs. This device generated up to 350 nW at a temperature difference of 20 K with only two pairs of TE legs. These works present TiS_2_ as one of the best TMDs for flexible, nontoxic, and room-temperature TE materials in terms of experimental *zT*.^120^

Finally, TaS_2_ with covalently bonded organic groups was investigated by Wang et al.^121^ This process improved the *zT* of the material by 10-fold compared to neat TaS_2_, reaching a PF of 340 μW m^–1^ K^–2^, which is the best-reported result for TMD composites. However, the high thermal conductivity limits the *zT* to 0.04. As reviewed, the TMD family has experienced steep progress over the last year, especially regarding PF, and these materials hold great potential for sustainable and performing room-temperature TEs.

### Black Phosphorus (BP)

Black phosphorus has been known in bulk since 1914. However, it has recently reemerged as a 2D material owing to its layered structure.^122^ 2D black phosphorus is a p-type monatomic 2D semiconductor composed of atomic layers stacked by Van der Waals forces. This structure allows the generation of few-layer and monolayer BPs via liquid-phase exfoliation (LPE). Exfoliation of the BP enables modification of the band gap, which increases with decreasing the number of layers. The thermoelectric properties of BP have been recently explored, making it a candidate for nontoxic flexible TE materials.^123^

2D BPs are truly novel materials, and most of the recent literature on the thermoelectricity of 2D BPs consists of theoretical works.^124,125^ Theoretical studies have predicted a Seebeck coefficient of over 300 μV K^–1^ and a *zT* of up to 1.2 at 500 K.^125,126^ Furthermore, the TE properties are highly anisotropic in layered BP, being the highest along the armchair direction as the thermal conductivity is noticeably lower. More recently, Zeng et al. reported a study on the TE properties of BP, experimentally demonstrating the anisotropic properties of this material. They obtained a *zT* of 0.043 in the armchair direction, whereas the *zT* in the zigzag direction was 5.5 times lower, i.e. a *zT* of 0.0075.^127^

At the experimental level, the greatest challenge in BP is to achieve stable monolayers; however, some recent reports have demonstrated the synthesis of monolayers.^128^ For example, Novak et al. obtained BP flakes via ball milling and red-phosphorus filtering. After this processing, the BP was mixed with PEDOT:PSS to improve its TE performance. The composite reached the highest PF when a 2 wt % of BP was mixed with PEDOT:PSS with a value of 36.2 μW m^–1^ K^–2^, representing an increment of 2.09 times compared to neat PEDOT:PSS.^129^

## Conclusions

In this work, recent advances in green TE materials for near-room-temperature applications, and examples of such materials in flexible and printed devices are reviewed. In particular, we focus on MOFs, COFs, MXenes, CuI, TMDs, black phosphorus, and their composites. The first two materials are organic or hybrid, whereas the others are pure inorganic. Table 1 shows a compilation of the literature reviewed in this study.

**Table 1:** Comparative between Different Works Reviewed in This Publication

material	PF (μW m^–1^ K^–2^)	*zT*	ref
Zn-HAB	0.344		(42)
Zr-MOF + PANi	664		(45)
Ni_3_(HITP)_2_ + CNT	24.86	0.0012	(47)
Ni-THT + SWCNTs	98.1		(49)
M-UiO-66 + PEDOT + SWCNT	27.9		(48)
MOF/SWCNT		0.02	(50)
F-COF + iodine	0.063		(58)
Mo_2_TiC_2_T_*x*_/Nb_2_CT*_x_*	13.26/11.06		(80)
Ti_3_C_2_T_*x*_ + KOH	44.98		(81)
Ti_3_CAlC_2_ + hexamine	33		(82)
Bi_2_Te_3_ + Mo_2_C	570	0.25	(85)
Ti_3_C_2_T_*x*_ + SnTe	2000		(90)
MXene + GeTe	40	1.12	(88)
Ti_3_C_2_T_*x*_ + SnSe		0.93	(89)
Bi_2_Te_2,7_Se_0,3_ + Ti_3_C_2_T_*x*_	1.49 × 10^3^	0.68	(86)
Ti_3_C_2_T_*x*_ + BST		1.3	(87)
SrTi_0,85_Nb_0,15_O_3_	3000	0.9	(96)
MoS_2_/NbSe_2_	0.537/0.035	5.4 × 10^–3^/1.36 × 10^–3^	(107)
TiS_2_	540	0.04	(111)
TiS_2_	1700	0.7	(112)
WS_2_ + rGO	17.4		(113)
MoS_2_ + Ag	30.3		(114)
MoS_2_ + Cu	125		(115)
MoS_2_ + Al	122		(116)
TaS_2_	340	0.04	(121)
TiS_2_ + hexylammonium		0.28	(117)
TiS_2_ + fullerene	375	0.3	(120)
Cu_2_S	10.1	1.1	(68)
Cu_2_S + Pb		2.03	(69)
Cu_2_S + PEDOT:PSS	20.3		(70)
BP + PEDOT:PSS	36.2		(129)
CuI	375	0.21	(71)
CuI	470		(72)
CuI	2400	1.12	(73)
CuI + Tb		0.28	(74)

Organic and hybrid materials are excellent options for flexible TEGs and all the works reviewed in the field of organic materials used nontoxic elements. However, their performance is lower than that of pure inorganic materials. From the organic materials reported, the most promising are COFs, as theoretical studies predict figures of merit close to 1, although the performance shown in experimental works is still much lower.

Within inorganic materials, MXenes are a great choice for composites to fabricate flexible and printable TEGs. However, the performance of the neat materials is low. Advances in the inorganic realm are more significant for metallic chalcogenides. These materials reach a *zT* of around 1.1, which is very close to the milestone of 1.5 suggested for TEs to be competitive with other renewable energy sources.^15^ Unfortunately, this high *zT* is reached at higher temperatures than the maximum *zT* achieved by organic materials. It is remarkable that many works within the metal chalcogenides family presented top TE performance among inorganic materials, but those top-performing materials rely on the use of toxic Se, which makes them unsuitable for green applications. The best result found in the literature is Pb-doped Cu_2_S that reaches a figure of merit of 2.03. However, the use of Pb, even at low concentrations, renders this compound far from green.

γ-CuI has recently emerged as a transparent TE that can achieve a high performance at low temperatures. The best result found in the literature corresponded to a *zT* of 1.12 at only 360 K, which is an outstanding result compared with the other materials listed in this review. Furthermore, this material is flexible and transparent, making it suitable for wearable devices.

Finaly, TMDs and BPs are barely studied materials, but theoretical studies show promising TE performances (*zT* up to 1.8 for TMDs). However, their experimental performance is still far from those predictions, which suggests a major opportunity for the field of TEs. The potential of TMDs was already partially fulfilled by TiS_2_, which stood out with an impressive experimental *zT* = 0.7 around room temperature.

From the reviewed literature, future trends in flexible TE materials are mainly oriented toward composites. The best performance, along with flexibility and printability, was achieved by combining different materials in synergy. In this context, MOFs and COFs are promising because their properties can be easily tuned. Furthermore, their organic nature makes them perfect candidates for green applications. Among inorganic materials, CuI is the most promising option owing to its high performance at low temperatures (*zT* > 1), nontoxicity, and abundance.
